# Metal Dyshomeostasis and Their Pathological Role in Prion and Prion-Like Diseases: The Basis for a Nutritional Approach

**DOI:** 10.3389/fnins.2017.00003

**Published:** 2017-01-19

**Authors:** Mattia Toni, Maria L. Massimino, Agnese De Mario, Elisa Angiulli, Enzo Spisni

**Affiliations:** ^1^Department of Biology and Biotechnology “Charles Darwin”, Sapienza UniversityRome, Italy; ^2^National Research Council (CNR), Neuroscience Institute c/o Department of Biomedical Sciences, University of PadovaPadova, Italy; ^3^Department of Biomedical Sciences, University of PadovaPadova, Italy; ^4^Department of Biological, Geological and Environmental Sciences, University of BolognaBologna, Italy

**Keywords:** metal dyshomeostasis, Mediterranean diet, transmissible spongiform encephalopathies, Alzheimer's disease, Parkinson's disease, synucleinopathies, prion, synuclein

## Abstract

Metal ions are key elements in organisms' life acting like cofactors of many enzymes but they can also be potentially dangerous for the cell participating in redox reactions that lead to the formation of reactive oxygen species (ROS). Any factor inducing or limiting a metal dyshomeostasis, ROS production and cell injury may contribute to the onset of neurodegenerative diseases or play a neuroprotective action. Transmissible spongiform encephalopathies (TSEs), also known as prion diseases, are a group of fatal neurodegenerative disorders affecting the central nervous system (CNS) of human and other mammalian species. The causative agent of TSEs is believed to be the scrapie prion protein PrP^Sc^, the β sheet-rich pathogenic isoform produced by the conformational conversion of the α-helix-rich physiological isoform PrP^C^. The peculiarity of PrP^Sc^ is its ability to self-propagate in exponential fashion in cells and its tendency to precipitate in insoluble and protease-resistance amyloid aggregates leading to neuronal cell death. The expression “prion-like diseases” refers to a group of neurodegenerative diseases that share some neuropathological features with prion diseases such as the involvement of proteins (α-synuclein, amyloid β, and tau) able to precipitate producing amyloid deposits following conformational change. High social impact diseases such as Alzheimer's and Parkinson's belong to prion-like diseases. Accumulating evidence suggests that the exposure to environmental metals is a risk factor for the development of prion and prion-like diseases and that metal ions can directly bind to prion and prion-like proteins affecting the amount of amyloid aggregates. The diet, source of metal ions but also of natural antioxidant and chelating agents such as polyphenols, is an aspect to take into account in addressing the issue of neurodegeneration. Epidemiological data suggest that the Mediterranean diet, based on the abundant consumption of fresh vegetables and on low intake of meat, could play a preventive or delaying role in prion and prion-like neurodegenerative diseases. In this review, metal role in the onset of prion and prion-like diseases is dealt with from a nutritional, cellular, and molecular point of view.

## Introduction

Many neurodegenerative diseases such as Alzheimer's disease (AD), synucleinopathies (including Parkinson's disease, PD), Huntington's disease (HD), amyotrophic lateral sclerosis (ALS), and frontotemporal dementia can be included under the definition of “prion-like disease” as they share some neuropathological features with the prion diseases (transmissible spongiform encephalopathies, TSEs). Overall, the prion and prion-like diseases have high social impact and cost as they include the most common age-related disorders. The proteins directly involved in prion-like diseases [Amyloid β (Aβ) for AD, tau for tauopathies, and α-synuclein (α-syn) for synucleinopathies] show similarities to prion protein in terms of the mechanism of seeding (Yamamoto et al., 2005; Nonaka et al., 2010) and spreading (Frost et al., 2009; Hansen et al., 2011) and of cell interaction modalities (Yamamoto et al., 2005; Nonaka et al., 2010). In the central nervous system (CNS), prion-like proteins can aggregate in an ordered “cross-β” assembly following a “nucleated growth” process and they can produce fibers that precipitate forming intracellular amyloid-like inclusions or extracellular amyloid deposits associated to neurodegeneration. Recently, Prusiner and collaborators demonstrated the ability of α-syn to serially propagate the infection both *in vitro* and *in vivo* systems defining it as a new prion protein (Prusiner et al., 2015; see Section “α-syn as New Human Prion” for more details). Prion proteins, α-syn and prion-like proteins (Aβ and tau) can directly interact with several metal ions affecting the conformation of the protein and its tendency to aggregate in oligomers and fibrils.

Epidemiological studies show that the occupational exposure to environmental metals such as manganese, copper, lead, iron, mercury, zinc, and aluminum is a risk factor for the development of neurodegenerative and prion-like diseases (Zayed et al., 1990; Rybicki et al., 1993; Gorell et al., 1999; Benedetto et al., 2009; Fukushima et al., 2010; Cannon and Greenamyre, 2011). On the other hand, metal ions and especially transition metals are key elements in organisms' life. They act like cofactors of many enzymes and are essential in cell respiration and metabolism due to their ability to accept or donate electrons passing from a reduced to an oxidized state (Barnham and Bush, 2014). However, transition metal ions can also be potentially dangerous for the cell as they can participate in redox reactions leading to the formation of reactive oxygen species (ROS) that can oxidize intracellular proteins, lipids, and nucleic acids. In particular, the radical-mediated oxidation of a protein can strongly affect its molecular structure by the generation of protein-protein linkages, the oxidation of amino acid side chains and even by the fragmentation of the polypeptide chain and the level of oxidized proteins increases during aging in many animals (for detail refer to Stadtman, 2006; Valensin et al., 2016).

For this reason, in the CNS any factor which induces a metal dyshomeostasis and the consequent production of ROS and cell injury may contribute to the onset of these neurodegenerative diseases. On the contrary, any factor able to reduce metal dyshomeostasis and to limit the production of ROS and free radicals can play a neuroprotective action. In addition to these general aspects, metal ions can have a more direct involvement in prion and prion-like neurodegenerative diseases for their ability to directly bind to prion and prion-like proteins and to affect the amount of amyloid aggregates.

The diet, source of metal ions and antioxidant agents such as polyphenols for the organism, is an aspect to take into account in addressing the issue of neurodegeneration. In recent decades, the food consumption in Western countries has undergone a rapid change that quickly led to the shift from a seasonal diet based on the consumption of fresh vegetable food and some meat derived from extensive farming to a massive consumption of packaged food and meat resulting from factory farms where animals are usually fed with a non-natural diet and subjected to drug treatments. The so-called mad cow disease is a striking example of how the change in farming method can contribute to the spread of prion diseases. In addition, the use of pesticides and herbicides in intensive agriculture determines the inclusion in the food chain of chemical substances that potentially can have harmful effects in the CNS even reacting with prion and prion-like proteins. Such changes in human diet lead to a variation in the nutrient intake that may affect the antioxidant and metal bioavailability in the CNS. Epidemiological data suggest that the Mediterranean diet (MeDi), based on the abundant consumption of fresh vegetables and on low intake of meat, could play a preventive or delaying role in prion and prion-like neurodegenerative diseases.

In this review, metal role in the onset of prion diseases (TSEs and synucleinopathies) and in AD, as an example of prion-like disease, is dealt with from a nutritional, cellular and molecular point of view.

## Prion protein as a model protein for prion diseases

The cellular prion protein (PrP^C^) is a highly conserved cell surface glycosylphosphatidylinositol (GPI)-anchored glycoprotein expressed in all mammalian tissues, particularly in the CNS (Harris, 1999). A conformationally-modified isoform of PrP^C^ called “Scrapie” (PrP^Sc^) is the major component of prions, the infectious particles at the basis of rare and inexorably fatal neurodegenerative disorders, called TSEs or prion diseases. TSEs develop on genetic, sporadic, or infectious grounds (Prusiner, 1998), and include bovine spongiform encephalophathy (BSE) in cattle, scrapie in sheep and goat, chronic wasting disease (CWD) in cervids and Creutzfeldt-Jacob disease (CJD) and Gerstmann-Sträussler-Scheinker syndrome in humans. TSEs are characterized by spongiform modifications in the brain, amyloid deposits with neuronal loss, and synaptic dysfunction. PrP^C^ and PrP^Sc^ though sharing the same primary structure and post-translational modifications (Stahl et al., 1993), are characterized by a different secondary structure. The β sheet-rich overall structure confers to PrP^Sc^ different physico-chemical and biologic features, including increased tendency to aggregate and resistance to proteolysis, the ability to self-propagate in a host organism and the acquisition of novel neurotoxic properties (Prusiner, 1998). The peculiarity of PrP^Sc^ is its ability to self-propagate in exponential fashion in cells, by acting as a model for the protein misfolding (Soto, 2012). PrP^C^ is tethered to the outside of the plasma membrane through its GPI anchor in plasma membrane subdomains called lipids rafts (Stahl et al., 1987) and it can shift to caveolae mediating signal transduction events (Toni et al., 2006). PrP^C^ NMR structure shows a flexible and disordered N-terminal domain (NT) (23–124 residues) and a C-terminal globular domain (CT) (125–228) that contains three α-helices, two short anti-parallel β-strands, and a short C-terminal tail (Zahn et al., 2000). This structure is stabilized by a single disulfide bond between Cys-179 and Cys-214 (human sequence). The NT contains five highly conserved proline- and glycine-rich octapeptide repeats (OR) (Harris, 1999). In spite of PrP^C^'s intimate involvement in TSE prion propagation, its function in cell physiology remains enigmatic, also because PrP-KO mice, in which the gene coding for PrPC was ablated (PrP-KO) by different gene-targeting strategies, showed no defects in embryonic and postnatal development and no behavioral alterations (Büeler et al., 1992; Manson et al., 1994; Mallucci et al., 2002) and only marginal phenotypes under normal conditions were observed in these mice (Criado et al., 2005; Nazor et al., 2007). These phenotypes could be due to the activation of compensatory mechanisms that hide the PrP-KO phenotype under physiological conditions and makes it detectable only under specific stress conditions or during aging (for a review see Ref. Peggion et al., 2016). However, several functions have been attributed to PrP^C^ (Peggion et al., 2016), some of which related to the ability of this protein to bind transition metal ions.

## Prion protein binds transition metals

Several metal ions are able to bind with different affinities to the PrP^C^, affecting its conformation and tendency to aggregate and influencing PrP^C^/PrP^Sc^ conversion. The OR region of PrP^C^ is the main metal binding site that can interact with Cu^2+^, Zn^2+^, and Mn^2+^ (Jackson et al., 2001; Walter et al., 2007; Brown, 2011) and it confers high stability to the protein (Benetti et al., 2014).

At physiological pH, PrP^C^ is able to bind four Cu^2+^ ions with high affinity in specific sites of OR region (Brown et al., 1997; Thompsett et al., 2005) and two additional Cu^2+^ at residues 96 and 111 with lower affinity (Jones et al., 2004). Many studies suggest the involvement of PrP^C^ in copper homeostasis (Brown et al., 1997; Wong et al., 2001a) as extracellular Cu^2+^ ions stimulate PrP^C^ endocytosis (Pauly and Harris, 1998; Brown and Harris, 2003), particularly at the pre-synaptic membrane (Vassallo and Herms, 2003) and regulate PrP^C^ expression (Toni et al., 2005).

PrP^C^ has been associated with metal-dependent enzymatic functions (Brown et al., 1999; Rachidi et al., 2003) and, consequently, with the enhancement of cell anti-oxidant potentials (White et al., 1999; Mitteregger et al., 2007; Peggion et al., 2016). Recently, a Cu^2+^-dependent neuroprotective role of PrP^C^ by mediating N-methyl-d-aspartate (NMDA) receptor-nitrosylation (Gasperini et al., 2015) has been demonstrated.

Experimental evidence supports the involvement of copper not only in PrP physiology but also in its pathology. In fact, the binding of copper to PrP would make the protein a relatively easy target of metal-catalyzed oxidation and it would lead to structural modifications that favor the PrP^C^–PrP^Sc^ conversion and the following protein aggregation. Experimental evidence shows that both histidine and methionine residues can be oxydated and involved in these events (Requena et al., 2001; Colombo et al., 2009). Currently available data are sometimes conflicting and does not allow a clear interpretation of Cu^2+^ role in prion disease. *In vitro* and *in vivo* experiments show that the addiction of Cu^2+^ induces the conversion of PrP^C^ into PrP^Sc^, increases the protease resistance and the infectivity of the protein, and overall accelerates prion disease (Pauly and Harris, 1998; Qin et al., 2000; Quaglio et al., 2001; Kuczius et al., 2004; Kim et al., 2005; Canello et al., 2012), while Cu^2+^ chelation delays the beginning of the disease (Sigurdsson et al., 2003; Siggs et al., 2012). On the other hand, similar experiments indicate that the presence of Cu^2+^ inhibits conversion of PrP^C^ into PrP^Sc^ and consequently its accumulation, delaying the onset of the disease in infected cells (Hijazi et al., 2003; Bocharova et al., 2005; Orem et al., 2006; Mitteregger et al., 2009). One possible explanation of these contrasting results comes from *in vitro* studies suggesting that Cu^2+^ ions can exert different effects on PrP^C^/PrP^Sc^ conversion depending on whether the template is constituted by soluble recombinant/purified PrP proteins or by preformed fibrils (Liu et al., 2007). Moreover, it has been reported that point mutations in PrP^C^ gene may cause coordinational changes at the copper site, favoring the conversion of PrP^C^ into PrP^Sc^ (D'Angelo et al., 2012; Giachin et al., 2015). Other possible explanations come from analyses of the ecosystems that support defined clusters of sporadic TSE. These studies suggest that Cu ions, such as Zn ions, can exert different effects on PrP^C^/PrP^Sc^ conversion depending on whether these transition metals are free or bound to scavenger co-factors in the CNS (Purdey, 2000).

TSEs are characterized by dys-metal homeostasis and by increased oxidative stress. Elevated levels of reactive oxygen intermediate species, nitric oxide and lipid peroxidation markers were detected both in the brains of mice infected with scrapie strains (Wong et al., 2001b) and in sporadic CJD frontal cortex homogenates (Freixes et al., 2006). The continuous conversion of PrP^C^ into PrP^Sc^ deprives neurons from the control of metal balance. However, whether the imbalance of metals is the cause or the consequence of TSEs is not yet known. PrP^C^ protein is able to bind Mn^2+^ at His96 and in the C-terminal region between residues 91 and 230 (Treiber et al., 2007; Brazier et al., 2008), protecting cells against Mn^2+^-induced oxidative stress (Choi et al., 2007). Manganese induces spontaneous PrP^Sc^ conversion (Brown et al., 2000) and increases the infectivity of PrP^Sc^ in cultured cells (Davies and Brown, 2009), while Mn^2+^ chelation decreases the amount of PrP^Sc^ present in infected mice brains (Brazier et al., 2010). Mn^2+^ levels are increased in blood and brain samples from subjects affect by BSE, scrapie, CJD, and from experimentally infected mice. Moreover, the increased manganese levels were inversely correlated to copper concentrations (Wong et al., 2001c; Thackray et al., 2002).

The influence of the environment on prion diseases is supported by studies indicating a geographical increase of CJD prevalence in people who have lived in a region of Slovakia where manganese is a major pollutant. High Mn^2+^ levels and increased Mn^2+^/Cu^2+^ ratios were observed in these CJD brains (Slivarichová et al., 2011). The link between Mn^2+^ and prion infection was also evidenced at the cellular level (Pass et al., 2015).

PrP^C^ binds also Zn^2+^ regulating its homeostasis, although with lower affinity and at low concentrations than Cu^2+^. Zinc, as copper, increases endocytosis of the prion protein causing the metal internalization and its elimination from the synaptic cleft. It has been also speculated that PrP^C^ acts as a sensor to monitor Zn^2+^ extracellular levels that may trigger a PrP^C^-induced signaling (Rana et al., 2009). Moreover, PrP^C^ may affect Zn^2+^ uptake via α-amino-3-hydroxy-5-methyl-4-isoxazolepropionic acid (AMPA) receptors (Watt et al., 2013). Studies conducted on several PrP^C^-peptides demonstrate that Zn^2+^ induces the increase of PrP^Sc^ aggregation while in the presence of full length PrP^C^, Zn^2+^caused a reduction of PrP^Sc^ deposition (Jobling et al., 2001; Kenward et al., 2007).

PrP^C^ contributes to maintaining iron homeostasis in the brain by regulating the iron uptake through the interaction with transferrin receptor pathway (Waheed et al., 2002) and by working as a ferric reductase in the transport of Fe^3+^ from endosomes to the cytosolic ferritin (Singh et al., 2015). Iron dyshomeostasis involves an incorrect nerve myelinizzation, altered neurotransmission, and affect bioenergetic processes resulting in neuronal death.

In sporadic-CJD brains, iron is sequestered in heat- and SDS-stable ferritin-PrP^Sc^ protein complexes, inducing an iron deficiency phenotype (Singh et al., 2009). So, despite the increased redox-activity of iron, its sequestration in complex aggregate proteins leads to a functional iron deficit. Moreover, Basu et al. (2007) demonstrated that the exposure to a source of reduced iron, such as inorganic ferrous chloride (FeCl_2_) induced the conversion of PrP^C^ to PrP^Sc^ implicating a role for this metal in the generation and propagation of PrP^Sc^.

## Synucleinopathies as prion diseases

The term “synucleinopathies” defines a group of human neurodegenerative disorders characterized by the presence of amyloidogenic α-synuclein (α-syn) inclusions that can occur in neurons and glia cells of the central and peripheral nervous system. These high social impact diseases include PD, Parkinson's disease dementia (PDD), dementia with Lewy bodies (DLB), multiple system atrophy (MSA), and a number of less well-characterized neuroaxonal dystrophies (Goedert, 2001).

Patients affected by synucleinopathies can show both motor and non-motor symptoms. PD is a progressive neurological disorder characterized by depression, anosmia, sleep disturbances, bradykinesia, resting tremor, rigidity, and postural instability (Fahn, 2003). Dementia is a frequent complication of PD characterized by deficits in attention, recognition memory and visual perception, by sleep disturbances, hallucinations and paranoid ideas (Perry et al., 1990; McKeith and Mosimann, 2004; Vann Jones and O'Brien, 2014; Garcia-Ptacek and Kramberger, 2016). MSA (also previously referred as Shy–Drager syndrome, olivopontocerebellar atrophy, and striatonigral degeneration) is characterized by muscle rigidity, tremor, cerebellar dysfunctions that include ataxia, and impairment of the autonomic nervous system (Goedert, 2001).

The amyloidogenic α-syn inclusions are intracellular proteinaceous bodies containing aggregates of hyperphosphorylated α-syn rich in β-sheets (Baba et al., 1998; Wakabayashi et al., 1998; Spillantini, 1999; Fujiwara et al., 2002) that are called Lewy bodies (LBs) and Lewy neurites (LNs) in PD, PDD, and LDB (Spillantini et al., 1997), glial cytoplasmic inclusions in MSA (Gai et al., 1998), and axonal spheroids in neuroaxonal dystrophies (Newell et al., 1999). A causative role for α-syn in the development of synucleinopathies has been established in PD via the discovery of mutations in the α-syn gene *SNCA* (A30P, E46K, H50Q, G51D, and A53T) resulting in autosomal-dominant PD (Kruger et al., 1998; Zarranz et al., 2004; Appel-Cresswell et al., 2013; Lesage et al., 2013; Proukakis et al., 2013; Pasanen et al., 2014).

**Figure 1:**
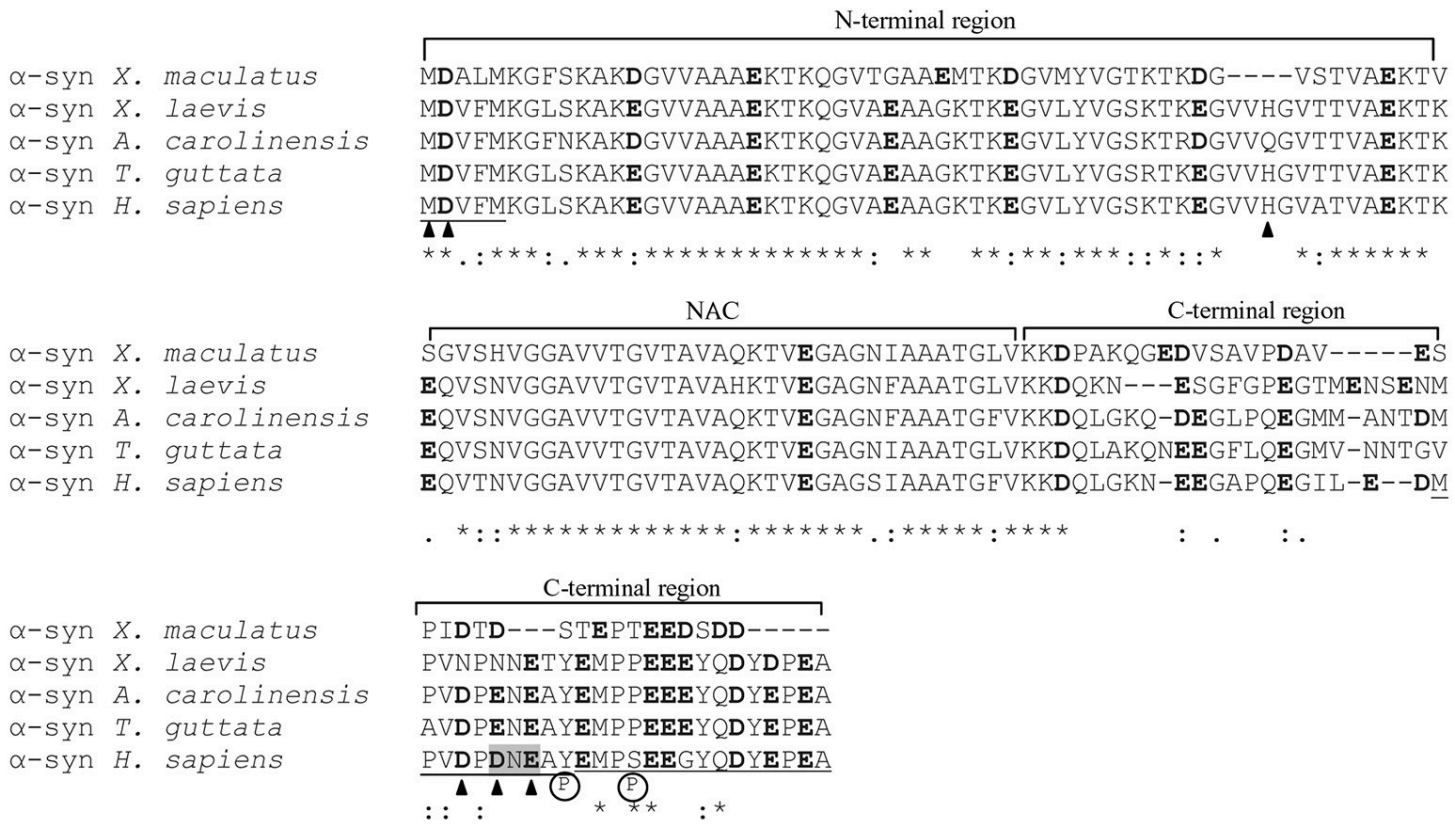
**Alignment of the α-syn amino acid sequences of representative species of teleost fish (***Xiphophorus maculatus***, accession number XP_005812724), amphibians (***Xenopus laevis***, NP_001080623), reptiles (***Anolis carolinensis***, XP_003221349), birds (***Taeniopygia guttata***, NP_001041718), and mammals (***Homo sapiens***, NP_001139526)**. Sequences were aligned with Clustal Omega (http://www.ebi.ac.uk/Tools/msa/clustalo/). Asterisks indicate identity of amino acids; double dots indicate amino acids with the same polarity or size; dots indicate semiconserved substitutions. Cu^2+^ binding sites are indicated by arrowheads, Cu^+^ binding regions are underlined, manganese, and iron binding sites are highlighted in gray and Ca^2+^ binding regions are double underlined. Negatively charged residues are indicated in bold characters. Circled P letter indicates residues whose phosphorylation increases the α-syn binding affinity for Cu^2+^, Pb^2+^, and Fe^2+^.

α-syn belongs to synuclein family together with β- and γ-syns. These proteins are particularly expressed at the level of the central (α- and β-syn) and peripheral (γ-syn) nervous system. In the CNS, α-syn is expressed in both neurons and glial cells and in neurons it is mainly localized in the cytoplasm and in presynaptic terminals, but a nuclear localization has been also reported (Vivacqua et al., 2011). The syn family members were sequenced in species representative of all vertebrates and the comparative analysis of amino acid sequences suggests that syns are evolutionarily conserved and fulfill important physiological functions (Toni and Cioni, 2015; Toni et al., 2016; Figure 1). However, the exact physiological roles of these proteins have not been fully clarified yet.

Human α-syn is a naturally unfolded protein composed of 24 negatively charged residues (Asp and Glu) that belongs to the class of “intrinsically disordered proteins” (Uversky et al., 2008; Figure 1). Its primary structure can be subdivided in the N-terminal region (NT) (1–60), the NAC (non-amyloid component) segment (61–95), and C-terminal region (96–140) (CT) (Figure 1). As in the case of the prion protein PrP^C^/PrP^Sc^, α-syn can exist in different conformations in the cell, passing from an unstructured conformation in solution to a high α helix percentages (63–71%) when linked to the phospholipid vesicles (Davidson et al., 1998) or to a major anti-parallel β-sheet conformation when aggregated into fibrils (Weinreb et al., 1996; El-Agnaf et al., 1998; Narhi et al., 1999). The NT can assume α-helix conformation binding to lipid membranes (Davidson et al., 1998) and from these features probably depend the involvement of α-syn in vesicle trafficking (Burré et al., 2010), neurotransmitter release and synaptic plasticity (Abeliovich et al., 2000; Murphy et al., 2000). The NAC segment is a highly hydrophobic region containing the amino acid sequence GVTAVAQKTVE capable of acquire antiparallel β-sheet conformation responsible for the aggregation and precipitation of α-syn proteins in insoluble fibrils (Narhi et al., 1999). The CT is an unstructured negatively charged region able to bind metal ions and proteins that probably acts as a scaffold for the recruitment of additional protein to the membrane (Eliezer et al., 2001).

## α-syn as a new human prion

**Figure 2:**
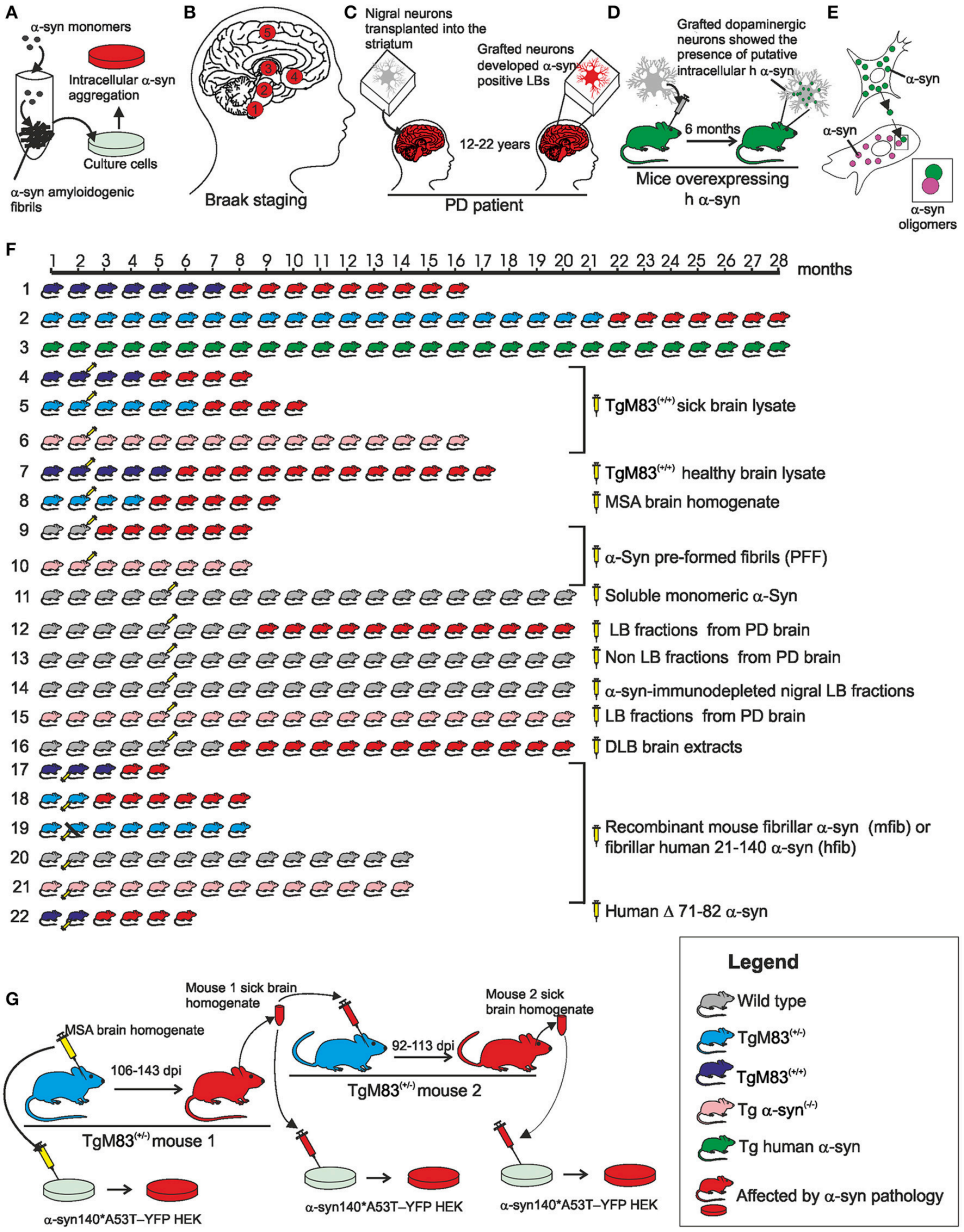
**Schematic overview of the main evidence in support of the prion nature of human α-syn. (A)** *In vitro* experiments, recombinant α-syn monomer can generate amylodogenic fibrils able to infect cultured cells. **(B)** In the idiopathic PD the formation of proteinaceous inclusion bodies begins in the dorsal motor nucleus of the vagus nerve and advances from there essentially upwards through susceptible regions of the medulla oblongata, pontine tegmentum, midbrain, and basal forebrain until it reaches the cerebral cortex (circled numbers refer to the synuclein progression in the CNS). **(C)** 12–22 years after transplantation into the striatum of individuals with PD, grafted nigral neurons developed α-syn positive LBs that stained positively also for ubiquitin providing evidence that the disease can propagate from host to graft cells. **(D)** Dopaminergic neurons extracted from the ventral mesencephalon of E12.5 C57BL/6 mice were grafted to the striatum of 6-week old transgenic mice overexpressing h α-syn. After 6 months, the grafted dopaminergic neurons showed putative intracellular h α-syn positive punctae demonstrating *in vivo* transfer of α-syn between host cells and grafted dopaminergic neurons. **(E)** Cell-produced α-syn is secreted via an exosomal calcium-dependent mechanism. The extracellular α-syn is taken up by cells through endocytosis and inside the cell interacts with intracellular α-syn forming dimers. **(F)** (1) Homozygous TgM83^+/+^ mice expressing A53T h α-syn in CNS neurons developed intracytoplasmic neuronal α-syn inclusions and a severe and complex motor impairment leading to paralysis and death starting from the 8th month; (2) hemizygous TgM83^+/−^ mice developed the same symptoms between 22 and 28 months of age; (3) Tg mice that express wild type h α-syn developed no motor impairment and revealed normal neuropil staining pattern expected for the protein up to the age of 28 months; (4) TgM83^+/+^ mice inoculated with brain homogenates from sick 12 or 18 month-old TgM83^+/+^ mice showed the characteristic motor clinical signs of illness after 97 days post-inoculation (dpi); (5) bigenic Tg mice (M83^+/−^; *Gfap*-luc) inoculated with brain homogenates from spontaneously ill 10 month-old TgM83^+/+^ sample showed symptoms of synucleinopathies 160 dpi; (6) C57B1/6S Δα-syn mice (presenting a deletion of the α-syn locus) inoculated with brain homogenates from sick, 12 or 18 month-old TgM83^+/+^ mice, show no signs of disease and were still alive and healthy 14 months post-inoculation; (7) brain homogenate from healthy 2 month-old TgM83^+/+^ inoculated in TgM83^+/+^ mice did not induce an acceleration in the onset of α-syn pathology in TgM83^+/+^ mice; (8) Tg mice (M83^+/−^; *Gfap*-luc) inoculated with brain homogenate from two independent confirmed cases of MSA began to exhibit signs of neurologic illness, most commonly ataxia and circling behavior, at about 90 dpi; (9) WT C57BL6/C3H mice inoculated with synthetic pre-formed α-syn fibrils (PFF) obtained *in vitro* from recombinant mouse or h α-syn progressively developed α-syn cytoplasmatic accumulation at 30 dpi that evolved in dense perinuclear LB-like inclusions by 90 and 180 dpi; (10) α-syn^−/−^ mice inoculated with PFF did not develop α-syn deposits; (11) no phosphorylated α-syn, ubiquitin or p62-positive pathology was observed in the brain of WT C57BL/6J mice inoculated with human or mouse α-syn monomer; (12) WT C57BL/6 mice inoculated with nigral LB-enriched fractions from post mortem PD brain resulted in progressive nigrostriatal neurodegeneration and diffusely α-syn accumulation within nigral neurons and anatomically interconnected regions by 4 up to 17 months post-inoculation; (13) WT C57BL/6 mice inoculated with nigral non-LB fractions from post mortem PD brain showed no nigrostriatal degeneration; (14) WT C57BL/6 mice inoculated with α-syn-immunodepleted nigral LB fractions from post mortem PD brain showed no phospho α-syn-positive cells and any evidence of pathology; (15) α-syn^−/−^ C57Bl6Sv129 mice inoculated with nigral LB fractions from post mortem PD brain did not produce any α-syn pathology or evidence of nigrostriatal lesions; (16) WT C57BL/6J mice brain injected with DLB insoluble fraction from post mortem PD brain showed in the 50% of cases immunopositive structures for anti-phosphorylated α-syn at 15 months post injection (17–21) recombinant full length mouse fibrillar α-syn (mfib) or fibrillar human 21-140 α-syn (hfib) were injected in the hind limb muscle of 2 month-old TgM83^+/+^ (17), of TgM83^+/−^ (18–19), of WT C3H/C57BL6 (20), or of α-*syn*^−/−^ (21) mice. The injected mice died or had to be killed due to paralysis within 57–88 (TgM83^+/+^) or 121 (TgM83^+/−^) dpi. Mice also developed α-syn inclusion pathology that was nearly indistinguishable morphologically in anatomic distribution from that seen in aged (>8 month-old) untreated TgM83^+/+^ mice. Four of the seven mice that had sciatic nerve transection showed no motor deficits 200 dpi (19). On the contrary, WT and α-syn^−/−^ mice developed no motor phenotype or α-syn pathology at 12 months post injection (20–21); (22) The injection of human Δ71–82 α-syn in TgM83^+/+^ mice resulted in delayed onset of disease (120 dpi) and incomplete penetrance of the pathology. **(G)** Inoculation with MSA brain homogenate in TgM83^+/−^ mice caused CNS dysfunction and the accumulation of large aggregates of phosphorylated α-syn and widespread astrocytic gliosis with mean incubation periods of 106–143 dpi (primary transmission). The inoculations in TgM83^+/−^ mice with brain homogenates from ill TgM83^+/−^ mice previously inoculated with MSA showed a shorter incubation period (92–113) (secondary transmission). The brain homogenate from MSA patients and from both serially infected TgM83^+/−^ mice were able to infect cultured cells. Figure drawings refers to the following references: (**A**; Desplats et al., 2009; Luk et al., 2009; Emmanouilidou et al., 2010; Nonaka et al., 2010; Volpicelli-Daley et al., 2011; Narkiewicz et al., 2014); (**B**; Braak et al., 2006); (**C**; Kordower et al., 2008; Li et al., 2008, 2010; Kurowska et al., 2011); (**D**; Hansen et al., 2011); (**E**; Emmanouilidou et al., 2010; Hansen et al., 2011); [**F**: 1–3 (Giasson et al., 2002); 4, 6, 7 (Mougenot et al., 2012); 5, 8 (Watts et al., 2013); 9, 10 (Luk et al., 2012); 11, 16 (Masuda-Suzukake et al., 2013); 12–15 (Recasens and Dehay, 2014); 17–21 (Sacino et al., 2014)]; (**G**; Prusiner et al., 2015).

A series of experimental evidence collected in the last 15 years supports the existence of a “prion like” mechanism in synucleinopathies according to which the α-syn pathology can spread between cells following neuroanatomical traits and the amyloidogenic β-syn can act as a template to guide the conversion of soluble, natively, unfolded α-syn to a conformationally-altered, aggregated form able to transmits from cell-to-cell. Recently, Prusiner and co-workers have defined the α-syn as a new human prion (Prusiner et al., 2015). Main experimental results are summarized in Figure 2 (for further details refer to the figure caption).

Pre-formed α-syn fibrils (PFF) rich in β-sheets with morphologies and structural characteristics similar to those extracted from LBs can be obtained *in vitro* from the polymerization of α-syn unstructured monomers. The α-syn fibrillation is a nucleated polymerization process in which the unfolded monomer undergoes self-assembly to form oligomeric intermediates (nuclei) at the onset of aggregation, followed by monomer accretion and fibril growth. A similar mechanism has been described in PrP aggregation process. PFF addiction to the culture medium induced the formation of protein aggregates in the cytoplasm of neuron cells (Figure 2A).

Studies of Braak et al. (2006) performed on numerous post-mortem cases of idiopathic PD support the capacity of pathological α-syn to spread in the human CNS suggesting the existence of a path of diffusion of the α-syn pathology from the dorsal motor nucleus of the vagus and in the anterior olfactory structure to the other brain regions (Figure 2B).

In order that a “prion-like” disease can be spread in the CNS, the causative agent must be transferred from one cell to another to induce the formation of amyloidogenic inclusions.

Monomeric and oligomeric forms of α-syn have been detected in human body fluids (plasma, saliva and cerebrospinal fluid; Borghi et al., 2000; El-Agnaf et al., 2003, 2006; Tokuda et al., 2006; Vivacqua et al., 2016) and *in vitro* and *in vivo* experiments showed that α-syn can be released from a donor cell, be taken up by a recipient cell, and then seed aggregation of endogenous α-syn within the recipient cell (Figures 2C–E).

Transgenic (Tg) mice expressing wild type (WT) human α-syn (h α-syn), or mutant A53T h α-syn M83 mice have been employed to provide compelling evidence for the detrimental role of α-syn inclusion formation in CNS neurons (Figure 2F). These studies highlight how the expression of the mutant A53T h α-syn in mice leads to neurodegeneration by promoting the formation of filamentous inclusions (Figures 2F1–3).

In order to further investigate whether α-syn can be spread with a “prion like” mechanism, several studies have been conducted based on the experimental approach used in the study of prion disease transmission: solutions containing α-syn protein of various origins have been administered to cell cultures or injected in WT or Tg mice to evaluate whether such administration may induce the formation of LB-like inclusion (Figures 2A,F4–21,G). Both recombinant α-syn (in the form of monomers, oligomers or fibrils; Figures 2F9,10,16–21) and brain homogenate from murine (Figures 2F4–7) or human subjects affected by α-syn pathologies (Figures 2F8,11–15) have been used.

On the whole these studies suggest that the injection of a lysate containing pathological α-syn in the brain of WT mice or Tg A53T h α-syn mice, or even in the hind limb peripheral nerve of Tg A53T h α-syn mice induces/accelerates the onset of the disease depending on the amount of pathological α-syn administrated and that α-syn expression in the host animal is necessary for the development of the pathology. Finally, the serial propagation of the infectivity in α-syn pathology, a prerequisite for a prion agent, was demonstrated by primary and secondary transmission in TgM83^+/−^ mice using pathological α-syn from MSA brain homogenates (Figure 2G). Collectively, these studies strongly support the prion nature of the pathological h α-syn.

## Role of metals in synucleinopathies

The involvement of metals in synucleinopathies is suggested by epidemiological studies showing a positive correlation between the occupational exposure to specific metals and the onset of PD and manganism, a disorder that shares many phenomenological features with PD, such as cognitive decline, psychiatric alteration and movement abnormalities (Zayed et al., 1990; Rybicki et al., 1993; Gorell et al., 1999; Benedetto et al., 2009; Fukushima et al., 2010). Elevated levels of several metal ions have been reported in the substantia nigra of people affected by PD (Gorell et al., 1997; Ayton and Lei, 2014). Furthermore, iron and aluminum have been detected in the LBs demonstrating the accumulation of metal ions in α-syn amyloidogenic aggregation (Dexter et al., 1991; Hirsch et al., 1991). In contrast, a lower concentration of copper was detected in brain regions of PD patients characterized by an accumulation of iron, if compared with controls (Dexter et al., 1991; Davies et al., 2014). Several lines of evidence support the possible involvement of iron in the pathogenesis of PD and specific reviews have been published on the subject (Götz et al., 2004; Mounsey and Teismann, 2012).

The risk of developing synucleinopathies increases with age. Interestingly, also the concentration of iron in the brain increases with age (Acosta-Cabronero et al., 2016; Pirpamer et al., 2016). A recent study has demonstrated an age-induced increase in the expression of both divalent metal transporter (DMT1) and α-syn in mice cells (Lu et al., 2016). This result describes a cellular scenario in which the cytoplasmic amount of both divalent metal ions and α-syn increase with age.

Many metal ions (mono, di and trivalent) can bind to α-syn by means of its negative charged residues, reducing their repulsion and affecting the protein tendency to aggregate in fibrils. This feature, also observed in PrP prion protein, can be closely related to the onset and development of synucleinopathies. Metal ions show different binding affinities for α-syn but the binding sites are similar for the majority of metal ions. Generally, divalent cations (such as Fe^2+^, Mn^2+^, Co^2+^, Ni^2+^, Ca^2+^, and Cu^2+^) bind to the CT of α-syn (Nielsen et al., 2001; Lowe et al., 2004; Binolfi et al., 2006) but binding sites for copper have been described also in NT and NAC segment (Camponeschi et al., 2013; Moriarty et al., 2014). Among metal ions, copper shows the highest binding affinity estimated in the μM range and it can bind to residues Met-1, Asp-2, His-50, Asp-119, Asp-121, and Glu-123 (Binolfi et al., 2006, 2012; Rodríguez et al., 2016; Figure 1). Manganese and iron can bind to residues Asp-121, Asn-122, and Glu-123 with an affinity in the range 1–50 mM (Golts et al., 2002; Binolfi et al., 2006) whereas the Fe^3+^ shows an affinity of 1.2 × 10 ^13^ M^−1^ (Peng et al., 2010).

The binding between metal ions and α-syn can be affected by pH-values (Drew et al., 2008) and protein modification such as phosphorylation (Lu et al., 2011), acetylation (Moriarty et al., 2014), and puntiform mutation (Drew et al., 2009). At acidic pH-values, Cu^2+^ binding sites shift toward the CT (Drew et al., 2008). The phosphorylation of residues Ser-129 and Tyr-125 increases the binding affinity for Cu^2+^, Pb^2+^, and Fe^2+^ (but not Fe^3+^) and determine a shift of the binding sites from the NT to CT (Lu et al., 2011). Among PD linked mutations, the A30P mutation favors the binding of Cu^2+^ to His50 whereas E46K, A53T did not seem to affect copper binding (Drew et al., 2009).

The rate of α-syn fibril formation *in vitro* was significantly accelerated by several divalent and trivalent metal ions such as Cu^2+^, Fe^3+^, Co^3+^, and Mn^2+^ (Uversky et al., 2001). The addition of Cu^2+^ to α-syn leads to a dynamically stable β-sheet conformation that serves as a nucleation point for a second- β strand (Rose et al., 2011) producing compact conformers toxic for the cell (Curtain et al., 2015). Moreover, μM concentrations of Fe^3+^ induce the formation of large SDS-resistant cytotoxic oligomers capable of forming pores in the lipid bilayer when added to the cells (Kostka et al., 2008). Interestingly, different metal ions induce the formation of α-syn oligomer**s** with different characteristics: Co^3+^ and Ca^2+^ produced annular oligomers (Lowe et al., 2004) whereas other metal ions (Ni^2+^, Fe^3+^, Cu^2+^, Mg^2+^, Cd^2+^, Zn^2+^) induced spherical oligomers (Nielsen et al., 2001; Figure 1D).

The potential involvement of α-syn in oxidative stress is an important issue as synucleinopathies, and in general neurodegenerative diseases, are associated with high levels of oxidative stress in the brain (Eskici and Axelsen, 2012; Schildknecht et al., 2013; Dixon and Stockwell, 2014). However, conflicting evidences on the causative or protective role of α-syn aggregation in ROS generation are available. On the one hand, it was shown that the oxidation of copper bound to α-syn can lead to the formation of hydrogen peroxide that exhibits a cytotoxic behavior in the cell (Lucas et al., 2010; Wang et al., 2010) promoting events proposed to be strongly related to the etiology of PD (Dell'Acqua et al., 2015) such as dityrosine crosslink (Lucas et al., 2010), dopamine oxidation (Meloni and Vasak, 2011), and methionine sulfoxidation (Ayton et al., 2013). On the other hand, a recent study showed that the copper/α-syn binding can exert a protective role against ROS. Pedersen et al. (2016) found that the levels of ROS and the rate at which they are generated, are significantly reduced when copper is bound to α-syn (and Aβ) particularly when the protein is in oligomeric and fibrillar form. These observations suggest that copper bound to the protein is less accessible to the solvent and therefore less capable of reacting with ascorbate, resulting in a reduced ROS formation, but further studies are necessary to elucidate this issue.

Recently, new data has been obtained on the interaction between iron and α-syn. In cell cultures the over expression of α-syn induced increased levels of intracellular iron and resulted in partial redistribution of iron from the cytoplasm to perinuclear inclusions (Ortega et al., 2016). Moreover, the expression of A53T mutant h α-syn aggravates Fe^2+^ mediated toxicity which results in an increased oxidative stress and DNA damage (Chew et al., 2011). Interestingly, intranasal treatment with desferrioxamine (DFO), a chelator widely used in clinical settings for the treatment of iron overload, down-regulated the expression of both α-syn and divalent metal transporter 1(DMT1; Guo et al., 2016).

Which exactly is the physiological function of the binding between metal ions and α-syn and whether it has an evolutionary significance is not yet completely clarified. The comparative analysis of α-syn amino acid sequences of representative species of teleost fish (*Xiphophorus maculatus*), amphibians (*Xenopus laevis*), reptiles (*Anolis carolinensis*), birds (*Taeniopygia guttata*), and mammals (*Homo sapiens*) shows that most of the negatively charged amino acids and sites able to bind copper, iron, and manganese ions in mammals are well-conserved among vertebrate species (Figure 1): Met-1, Asp-2 are conserved in all the five sequences analyzed, whereas, Asn-122, and Glu-123 are conserved in all tetrapoda species considered. Asp-119 is not conserved only in *X. laevis* in which it is substituted by Asn, His-50 lacks in *X. maculatus* and *A. carolinensis*, whereas Asp-121 is conserved only in *X. maculatus*. Interestingly, Tyr-125 and Ser-129, residues whose phosphorylation increases the affinity for Cu^2+^, Pb^2+^, and Fe^2+^, are perfectly conserved among the tetrapoda species analyzed.

Although there is variability in the number of syn isoforms in non-mammalian vertebrates (Toni and Cioni, 2015) and more data and detailed studies are necessary for a thorough analysis, the data here reviewed suggest that the binding of metal ions to α-syn may be involved in evolutionary conserved, as not yet clear, physiological functions and that the concomitant presence in the cytoplasm of high amount of α-syn protein and high metal ions concentrations makes the cell more prone to the formation of amyloid aggregates and the organism more susceptible to disease development.

The amount of metal ions in the brain undergo changes during the course of life (Acosta-Cabronero et al., 2016; Pirpamer et al., 2016), and the metal ions concentration may vary in the different brain regions (Ayton and Lei, 2014). Given the broad spectrum of effects of metal ions on the cell physiology, this means that in the course of human life the change in metal ions concentration can make the nervous system more prone and susceptible to the onset of neurodegenerative prion diseases. In this sense, the metal intake through the diet could influence the onset of prion and prion-like neurodegenerative diseases.

## Alzheimer's disease as a prion-like disease

Alzheimer's disease (AD) is a neurodegenerative disorder involving progressive cortical and hippocampal neuron loss, clinically characterized by progressive and irreversible cognitive deficits and behavioral alterations that affect memory, learning ability, and the quality of life. There are two distinct clinical manifestations of AD, familial and sporadic, both characterized by the aggregation of misfolded proteins, inflammation, and metabolic failure. Although AD is a genetically complex autosomal dominant disease, caused by mutations in the amyloid precursor protein (APP) gene, presenilin 1 (PSEN1), or presenilin 2 (PSEN2; Bertram et al., 2010), the majority of AD cases are sporadic. Pathologically, AD is characterized by amyloid deposits that consist of extracellular aggregated Aβ, neurofibrillary tangles (NFTs) composed by the hyperphosphorylated tau protein, and by neuronal losses (Hardy and Higgins, 1992). The Aβ peptides are produced by the cleavage of the APP through the so called “amyloidogenic pathway.” In this way, the sequential activity of β-secretase (BACE1; Cole and Vassar, 2008) and γ-secretase, a multiprotein complex that contains both PSEN1 or PSEN2 (Steiner, 2008), produces APP fragments of different length (39–42 amino acids) among which Aβ42 is considered particularly neurotoxic and more prone to self-aggregation than shorter peptides.

Research conducted over the past two decades have shown many similarities between Aβ and prion properties and for this reason Aβ is generally included among the prion-like proteins. Recently, comprehensive reviews on this fascinating subject have been published to which refer for in-depth details and specific references (Tatarnikova et al., 2015; Ugalde et al., 2016; Walker et al., 2016). Aβ, like PrP, may undergo conformational changes assuming a tertiary structure rich in β sheets that promotes the self-assembly of the protein in oligomeric and fibrillar aggregates with neurotoxic properties (Haass and Selkoe, 2007; Klein, 2013). Interestingly, insoluble Aβ seeds are relatively resistant to proteinase K (PK) whereas Aβ soluble fraction is largely PK sensitive and it loses its seeding activity after PK digestion (Langer et al., 2011).

Several experiments based on the intracerebral injection of AD brain homogenate in non-human primates and in transgenic mice and rats (Baker et al., 1993; Meyer-Luehmann et al., 2006; Rosen et al., 2012) showed an increase in the amount of Aβ deposits in the receiving brain that augmented with longer incubation time and with higher concentration of the injected homogenates. On the contrary, the use of both control brain homogenate and AD brain homogenate in which Aβ had been depleted by antibodies did not induce the Aβ accumulation (Meyer-Luehmann et al., 2006; Duran-Aniotz et al., 2014), suggesting that the pathological Aβ contained in the AD brain can be the promoting agent. In support of this hypothesis, the deposition of Aβ can be observed also after the injection of high concentrations of multimeric synthetic Aβ accompanied by long periods of incubation (Stöhr et al., 2012).

The deposits of seed-induced Aβ, initially found only in tissues near the site of injection, gradually propagate along axons to different brain regions with stereotypic temporal-spatial spreading patterns which are partly reminiscent of the spreading characteristics of prions. From this point of view, the deposition of the pathological protein in AD can be explained by the initial formation of protein seeds early in pathogenesis, followed by a prion-like spread of misfolding and aggregation events along the neuroanatomical pathways.

## Role of metals in AD

Although genetic, biochemical, and neuropathological data indicate that amyloid formation plays a central role in AD pathogenesis (Selkoe, 2001), the aetiopathology of this disease remains unclear. Compelling evidence suggest the existence of a correlation between the imbalance of metal homeostasis in the brain and the pathogenesis of AD. Transition metals could participate in AD pathogenesis by interacting with Aβ peptide, promoting its aggregation and facilitating ROS production and oxidative stress. Focal accumulation of Zn, Cu, and Fe might also deprive other brain tissues of these essential metals, leading to aberrant neuronal function. The total amount of copper (390 μM), zinc (1055 μM), and iron (940 μM) have been reported to be increased in AD brain as compared to normal age-matched samples (copper, 70 μM; zinc, 350 μM; iron, 340 μM; Lovell et al., 1998). At a cellular level, the analysis of the amount of metal ions in samples from AD patients revealed increased zinc and iron (Deibel et al., 1996; Bouras et al., 1997; Duce et al., 2010), decreased levels of copper (Bouras et al., 1997; Smith et al., 1997; Duce et al., 2010) and imbalances in aluminum, silicium, and mercury.

Concerning Zinc, Bush et al. (1994) provided the first biochemical evidence that this metal ion is able to bind Aβ and to cause its aggregation and precipitation initiating the plaque formation. Zinc also interferes with Aβ processing, by inhibiting γ-secretase activity and increasing PS1 expression (Lammich et al., 1999). Through the activation of tau-kinases, Zn can also impact on tau-related neurotoxicity (Mo et al., 2009). The dyshomeostasis of Zn in the brain can lead to an enhanced susceptibility to the excitotoxicity of glutamate and to oxidative stress (Oteiza et al., 1995; Takeda et al., 2004), being this metal an inhibitory neuromodulator of glutamate release in the hippocampus and antagonizing the catalytic properties of the redox-active transition metals.

Copper, like Zn, is synaptically released and acts as a potent mediator of Aβ aggregation (Mantyh et al., 1993). It binds with high affinity to an amino terminal tyrosine residue in Aβ inducing its oligomerization and neurotoxicity. Moreover, the elevated copper amount was detected in amyloid plaques. The cytotoxicity induced by the Cu-Aβ complex involves also oxidative stress events, as this complex catalytically generates hydrogen peroxide (Huang et al., 1999), through the reduction of Cu^2+^ to Cu^+^, accompanied with the oxidation of endogenous molecules such as thiols, cytochrome c oxidase, ascorbate and lipids (Turnbull et al., 2001; Puglielli et al., 2005). The resulting lipid peroxidation leads to the formation of peroxyl radicals, which once formed, can then be rearranged into compounds like malondialdehyde (MDA; Jomova and Valko, 2011) and 4-hydroxy-2-nonenal (HNE), detected at higher levels in AD brain and AD transgenic mouse models (Haeffner et al., 2005; Nelson and Alkon, 2005). However, although Cu^2+^ is increased in amyloid plaques, it is decreased in AD neuronal tissue which in turn could deprive Cu-binding proteins such as superoxide dismutase and ceruloplasmin and impairs their function. Copper also influences the fate of APP processing, given its participation in APP processing into non-amyloidogenic derivatives while its deficiency reduces Aβ degradation (Cater et al., 2008).

Concerning Fe, its deposition in neurons causes oxidative stress via the Fenton reaction, producing abnormalities in RNA, yielding a great reduction in protein synthesis and initiating several Fe-induced apoptotic signaling pathways (Salvador and Oteiza, 2011). Fe indirectly damages proteins such as Ca^2+^-ATPase, glutamate transporter, Na^+^/K^+^-ATPase as well as N-methyl-D-aspartate (NMDA) receptor, and lipids such as cholesterol ceramides and unsaturated fatty acids through the formation of hydrogen peroxide and the hydroxyl radical (Kaplán et al., 1997; Mark et al., 1997; Muñoz et al., 2011; Shinkyo et al., 2011). Elevated cellular Fe levels can cause cell death independently of ROS toxicity by the phenomenon known as ferroptosis, a type of Ras-related cell death pathway (Dixon et al., 2012), while its increased concentration at synapses may also leads to increased Aβ production by altering its processing. In fact iron can act by decreasing the protein furin, which modulates γ–secretase activity, and increased iron concentration favor γ–secretase activity and enhances the amyloidogenic pathway. Although metal dyshomeostasis in AD is primarily due to metal sequestration by amyloid plaques and NFTs, the disruption of the vesicular trafficking has also to be considered since it prevents metal ion containing vesicles to arrive at the axon terminal. The disruption of axonal transport affects also the mitochondrial transport preventing the replacement of the old organelles with the new ones and the production of the energy required for the reuptake of Cu/Zn from the postsynaptic neuron. The acquisition of knowledge of the role of metals in AD neurodegeneration has prompted the discovery of metal-chelating compounds (such as desferrioxamine and deferiprone) conjugated to nanoparticles favoring their passing through the blood-brain barrier that *in vivo* prevent the toxic actions of transition metals (Li et al., 2011). Presently, only one family of metal-binding agents, PBT2 [5,7-dichloro-2-((dimethylamino)-methyl)-8-hydroxyquinoline], is testing in clinical trials for the treatment of AD. PBT2 binds excesses of copper, zinc, and possibly iron in the brain, thereby diminishing the amount of amyloid plaque formation and relocating these metal ions to depleted cellular and neuronal compartments (Crouch et al., 2011).

## Mediterranean diet, prion and prion-like diseases, and neurodegeneration

The traditional Mediterranean diet (MeDi) is characterized by an abundant consumption of plant foods, a moderate intake of fish and wine, and low intakes of meats and dairy products, with extra-virgin olive oil as the main fat source. Adherence to the MeDi has also been associated with a lower risks of AD, PD, dementia, and cognitive decline (Scarmeas et al., 2006, 2009; Féart et al., 2009; Singh et al., 2014) while no epidemiological data are available for the rare sporadic CJD. Human epidemiological study, such as the Northern Manhattan Study have confirmed that MeDi is associated with a lower white matter hyperintensities (VMH) burden evaluated by magnetic resonance imaging, a marker of small vessel damage and neurodegeneration (Gardener et al., 2012). Many studies have demonstrated that MeDi is associated with a significant reduction in AD risk. Results based on observational studies (Valls-Pedret and Ros, 2013) and long-term randomized clinical trials have clearly shown that a correlation between MeDi and AD exists with strong level of scientific evidence (Valls-Pedret et al., 2015).

Higher MeDi adherence was also associated with reduced odds for PD, after adjustment for all covariates while on the contrary, lower MeDi adherence was associated with earlier PD age-at-onset (Alcalay et al., 2012).

In PD, there is considerable evidence for ROS-mediated damage in post mortem brain samples as well as in other tissues, even outside of the central nervous system. Oxidative damage to nucleic acids, lipids and proteins in both the brain and peripheral tissues in human PD has been clearly evidenced (Sanders and Greenamyre, 2013).

Even if the neurobiological basis of the relationship between the MeDi to brain health has not been elucidated yet, various specific nutrient components of this diet have been examined in relation to cognitive performance including dietary fatty acids, antioxidants, amount of fruits and vegetables consumption, vitamins (particularly B6, B12, and folate; Smith and Blumenthal, 2010). The MeDi is a very complex eating pattern, with a multitude of single components that could cause beneficial neuroprotective effects (Jacobs et al., 2009; Gotsis et al., 2015). In the midst of this multitude, plant polyphenols, which occur mainly in fruit, vegetables, and wine (Manach et al., 2004) have recently shown to possess beneficial effects with regard to overall health, as well as cognitive functions (Baur and Sinclair, 2006; Witte et al., 2014). Plant polyphenols represent an abundant class of plant secondary metabolites found in herbal-rich food and beverages, with no <8000 phenolic structures having been identified in plants (Tsao, 2010).

For example, oleuropein aglycone (OLE), the main polyphenol in the extra-virgin olive oil has clearly showed to ameliorate memory dysfunction, neuronal loss and neurodegenerative damages in AD animal models (Grossi et al., 2014). Dietary supplementation of OLE (50 mg/kg of diet), strongly improved the cognitive performance of young/middle-aged TgCRND8 mice, a model of Aβ deposition. In these mice, a reduced β-amyloid levels and plaque deposits was associated with a strong increase of autophagic markers expression and of lysosomal activity (Grossi et al., 2013). In human population, serum concentration of some phytochemicals, characteristic of the Mediterranean diet (such as lutein, zeaxantin, and β-carotene), were consistently related to better cognition functions in the Georgia centenarian study (Johnson et al., 2013).

Much effort has been undertaken in the way of understanding the neuroprotective effects of polyphenols, using both *in vitro* and *in vivo* models (Johnson et al., 2013). The molecular mechanisms of their neuroprotective actions can be classified as anti-inflammatory, antioxidant (free radical scavenging and metal chelation), anti-amyloid action, or through direct modulation of cell signaling pathways, such as metalloproteinases inhibition or their activity on transcription factors such as NF-kB (Gomes et al., 2008; Crascì et al., 2016). Their ability to simultaneously and synergistically modulate multiple molecular targets, suggests a greater potential also for therapeutic intervention in AD and PD (Grodstein et al., 2007; Johnson et al., 2008). Since transition metal and particularly iron have been clearly involved in neurodegenerative disorders and aging (Ward et al., 2014), in the next section, we focus on the free radical scavenging and metal chelation effects of some polyphenols.

## Polyphenols as metal chelators and free radical scavengers

Polyphenols are well-established metal chelators and some of them possess the ability to bind and chelate many different bivalent metals, such as Cu^2+^, Zn^2+^, and Fe^2+^ (Singh et al., 2008; Mandel et al., 2011). In this way, the rate of Fenton reaction directly diminishes and the oxidation caused by reactive hydroxyls radicals can be prevented (Perron and Brumaghim, 2009). Moreover, polyphenols decrease metal absorption by exerting their chelating activities also into the small intestine (Landete, 2012). In humans participating in the PAQUID cohort study, higher levels of polyphenols (flavonoids) were associated with ~50% reduction of the risk to develop dementia (Commenges et al., 2000).

In animal models, it has been clearly demonstrated that various polyphenols, capable to chelate divalent metals and with antioxidants activities, possess neuroprotective effects. Dairam et al. (2008) have observed that curcumin modulates iron in rat-brain homogenates. In a rat model of Parkinson's disease, curcumin has shown neuroprotective effects by decreasing neuron degeneration (Du et al., 2012). Epigallocatechin Gallate (EGCG) is the most effective antioxidant polyphenol in green tea and there is clear evidence that EGCG is very effective in metal chelation of Fe^2+^, Zn^2+^ and Cu^2+^ (Chan et al., 2016). In SH-SY5Y neuroblastoma cells, EGCG has exhibited stronger iron chelation compared to desferrioxamine (Reznichenko et al., 2006).

At the molecular level, different polyphenols have shown to inhibit Aβ42 fibril formation by directly interacting both with Aβ42 and transition metals. For example, glycosylated polyphenols (such as verbascoside present in the olive tree) and their esterified derivatives regulate metal-free and metal-induced Aβ42 aggregation and disaggregation at 50 μM concentrations (Korshavn et al., 2015).

EGCG, at 100 μM completely inhibited the formation of Aβ42 fibril and was capable to reduce the amount of fibrils present when EGCG was added demonstrating its ability of remodeling preformed fibrils (Chan et al., 2016). Curcumin, one of the principal polyphenols in turmeric (*Curcuma longa*) exhibited moderate metal chelation and antioxidant activity, and it is a known inhibitor of Aβ fibril formation *in vitro* (Ono et al., 2004).

Red wine is famous for its polyphenol content. The major polyphenols found in red wine extracts include resveratrol, quercetin, catechin, epicatechin, tyrosol, gallic acid, and procyanidins. Many different *in vitro* studies have described the potent free radical scavenging effects of red wine polyphenols, including direct scavenging of reactive oxygen and nitrogen species, such as peroxides, superoxide, the hydroxyl radical, and the peroxynitrite anion, as well as sequestering of highly redox-active metal ions (Wang and Brumaghim, 2011). Treatment of neuronal and astrocytic cell lines with these polyphenolic compounds suppressed ROS production and significantly improved cell viability (Martin et al., 2011, 2013). Resveratrol, mostly present in berries, grapes and wine, was protective in human neuroblastoma cells exposed to Aβ or to Aβ-metal complexes through its scavenging properties (Granzotto and Zatta, 2011). Several polyphenols present in wine have been demonstrated to protect mitochondria from ROS damages in *in vitro* studies. These include resveratrol, quercetin, anthocyanidins, and proanthocyanidins (Fernández-Moriano et al., 2015). Moreover, resveratrol administration, as a dietary supplement, significantly attenuates 6-hydroxydopamine-induced oxidative damage, and dopamine depletion in a rat model of Parkinson's disease (Khan et al., 2010).

Since resveratrol is able to penetrate the blood–brain barrier and exert strong neuroprotective effects, even at low concentrations, it has been used in a clinical trial that has currently completed phase II in 120 patients with possible AD diagnosis (NCT01504854). Ongoing trials are investigating the efficacy of a mixture of grape polyphenols in mild cognitive impairments (MCI) and moderate AD (i.e., NCT02502253).

To date, although some polyphenols can efficiently cross the blood-brain barrier, the literature suggests that single polyphenol may not attain concentrations within the brain that are sufficient to exert an effective metal chelation and free radical scavenger activities. Nevertheless, MeDi is characterized by a high consumption of many different polyphenols-rich foods and beverages that may exert their neuroprotective effects in a time-dependent manner. Epidemiological studies suggest that the activities of these complex mixtures of multitarget compounds could be protective and really effective at preventing or delaying prion-like diseases with a high prevalence in the population, such as AD and PD (Alcalay et al., 2012; Pelletier et al., 2015).

## Conclusions

Neurodegenerative diseases such as TSEs, synucleinopathies, AD, Huntington's diseases, and ALS belong to the prion and prion-like diseases, disorders in which specific proteins precipitate in amyloid aggregates in the nervous system as a result of conformational change. Among these diseases, AD and PD are disorders of concern for their high social impact and cost as they are the most widespread neurodegenerative diseases in the populations of Western European countries and the USA. Epidemiological studies and experimental evidence at the cellular and biochemical level show the influence of the dyshomeostasis of metals in the onset of such neurodegenerative diseases. It is noteworthy that different risks of developing AD and PD have been associated with different nutritional habitus. Among them, the Mediterranean diet was found to be the most protective, with significant reduced odds for PD and AD. This diet is characterized by a very high content of different multifunctional polyphenols capable to bind metals (transition metals in particular) and amyloid aggregates, acting by specific regulators of aggregation and cytotoxicity of metal-free and metal-associated prion and prion-like proteins. Many of these polyphenols can cross the blood-brain barrier and serve as specific and multitarget compounds capable to regulate ROS damages, inflammation and aggregation of misfolded proteins in neuronal tissues.

Having classified the major neurodegenerative diseases within the classes of prion and prion-like diseases implies that we must accept the idea that nutritional factors are capable to modify the mechanisms of neuronal damage induced by prion and prion-like proteins and that the Mediterranean diet can exert a protective role in these diseases.

## Author contributions

MT, MM, and ES drew the manuscript. MT, MM, AD, EA, and ES wrote the manuscript.

### Conflict of interest statement

The authors declare that the research was conducted in the absence of any commercial or financial relationships that could be construed as a potential conflict of interest. The handling Editor declared a past co-authorship with one of the authors ES and states that the process nevertheless met the standards of a fair and objective review.
